# Development of a Symptom Self-Management Guide for Older Chinese Americans Kidney Receiving Replacement Therapy

**DOI:** 10.1155/2024/2280296

**Published:** 2024-10-11

**Authors:** Carolyn Sun, Wing Cheung, Kathryn Corpuz, Jingjing Shang, Patricia W. Stone

**Affiliations:** ^1^Hunter College, Hunter-Bellevue School of Nursing, 425 E 25th St., New York, NY 10065, USA; ^2^New York University, 726 Broadway, 4th Floor, Suite 403, New York, NY 10003, USA; ^3^Columbia University School of Nursing, Center for Health Policy 560 W 168th St, New York, NY 10032, USA

## Abstract

**Aim:**

To assess the acceptability of a symptom self-management booklet among older Chinese Americans receiving kidney replacement therapy.

**Background:**

In previous work, we identified commonly occurring, bothersome symptoms and strategies used in this population to ameliorate symptoms. We used these data to develop a symptom self-management booklet in English, traditional, and simplified Chinese.

**Introduction:**

In the United States, the prevalence of kidney disease is 1.5 times higher in Asians compared to whites. With the many symptoms associated with this disease, self-management of symptoms would be particularly helpful.

**Methods:**

Seven older Chinese Americans receiving kidney replacement therapy and their caregivers were interviewed to assess the acceptability of the booklets. We reviewed participant feedback on content, graphics, and design, reading experience, suggestions for improvement, and health information sources using the inductive thematic method.

**Results:**

Overall, patients confirmed acceptability of these self-management booklets across all domains. *Discussion*. This study validated the booklet as a source of health information for older Chinese American patients with kidney disease, which some studies suggest are preferred to electronic materials or methods in this population. Health care providers can use the resultant booklets when caring for these patients to provide culturally sensitive information on self-management of symptoms. *Conclusion and Implications for Nursing*. These booklets provide a free resource tailored to an underserved population and may help nurses and nurse practitioners provide care with cultural humility. *Implications for Health Policy*. Embracing community-based participatory research, as was done in this study, can help create culturally appropriate patient education materials that empower patient symptom self-management and promote informative and culturally sensitive conversations between patients, families, and providers.

## 1. Background

Kidney failure related to chronic kidney disease (KFCKD) is the result of permanent kidney failure and is a progressive medical condition in which a person's life can be maintained only by long-term, intensive dialysis or kidney transplant, termed “kidney replacement therapy” (KRT). Patients with KFCKD are not only distressed physically and mentally by the burdensome treatment process but also by the many symptoms of the disease [1].

The prevalence of KFCKD in the Chinese American population is of special concern. The incidence of CKD is 1.5 times higher in the Asian American community compared to the Caucasian community [2]. The growth of the Asian population was the fastest among all race groups in the U.S. from 2000 to 2019, at a rate of 82.2% [3]. The greatest proportion of the Asian population in the U.S. in 2019 was an estimated 5.3 million Chinese of Asian descent [3]. With the rapid growth of Chinese Americans, it is reasonable to expect that the number of Chinese Americans with CKD will continue to expand.

Medicare beneficiaries with KFCKD who choose to continue dialysis therapy are ineligible for Medicare-funded hospice care, creating barriers to accessing palliative service [4, 5]. This policy disadvantages older Chinese American patients who rely heavily on Medicare, making management of symptoms a crucial skill; symptom self-management has been demonstrated to be an effect strategy for improving self-efficacy, medication adherence, and quality of life [6].

Conservative treatment (i.e., management of symptoms rather than initiating dialysis or opting for transplant) is considered a viable option that may increase quality of life in older patients that may not receive benefits that outweigh the burdensome nature of KRT [7]. However, this option frequently goes undiscussed as it can be perceived by patients as a failure to intervene [8]. The Individual and Family Self-Management Theory suggests that many factors influence whether a patient can practice self-management behaviors, including knowledge, cultural norms, and personal preferences. Those patients with beliefs consistent with the self-management behaviors as well as social support (including shared-decision making with their health care providers) are more likely to engage in recommended self-management behavior [9]. This idea of the provider and the patient discussing treatment plans and coming to an agreement resulting in improved patient medication and treatment taking behavior has been termed, “concordance” [10].

Chinese Americans are also disadvantaged because of language barriers. In the U.S., nearly 34% of Chinese Americans speak either Mandarin or Cantonese Chinese at home and 43% of foreign-born Asians had less than proficient English-speaking ability or did not speak English at all [11]. This limited English proficiency may result in low health literacy [12]. Compared to other racial/ethnic groups, Chinese with limited English proficiency reported the highest prevalence of low health literacy [13]. Low health literacy puts this group at a high risk for poor health outcomes [13]; for example, it may decrease adherence with self-management strategies [14]. Conversely, when using their native language to discuss health issues, Chinese Americans were found to have higher health literacy regardless of their English proficiency level [12].

To align with Healthy People 2030 (released in August 2020 by the U.S. Department of Health and Human Service (HHS)), strategies to promote health literacy in older Chinese Americans with KFCKD should be developed. According to Healthy People 2030, health literacy will be the central focus in the promotion of health and well-being of all in the next decade [15]. In addition to the prior national objectives for “eliminating health disparities, achieving health equity, and attaining health literacy to improve the health and well-being of all,” the new definition of health literacy used in Healthy People 2030 also emphasizes the importance of both personal and organizational health literacy to help individuals to “find, understand, and use information and services to inform health-related decisions and actions for themselves and others” at personal and organizational levels [15].

To promote health literacy in the older Chinese KFCKD population, one approach may be the use of printed health information materials, such as booklets, in their preferred language. According to a study investigating the preference of health information sources across various non-English speaking groups, brochures and emails were the preferred source of information [16]. When health education materials are translated, they must take into consideration Chinese cultural contexts and practices and make them readily available for this population [12].

The aim of this study was to develop an educational booklet for patients with KFCKD with evidence-based, culturally sensitive information about self-symptom management strategies and to evaluate its acceptance among older Chinese Americans with KFCKD. Creating the booklet content was done in stages. First, we identified the bothersome symptoms commonly experienced by this population and the self-management strategies they used for symptom amelioration. Then, strategies were confirmed through an umbrella review and a panel of experts (including a Traditional Chinese Medicine doctor, a physician, palliative care and adult gerontology nurse practitioners, and nurses) in a previously published study [17].

Using the content identified from this study, we created and tested a booklet to guide the self-management of symptoms in older Chinese Americans with KFCKD and MCC. It was hypothesized that the booklet would be acceptable to these target patients and that the key messages in the booklet would help enhance their skills in managing their symptoms.

## 2. Methods

The study was conducted in New York City (NYC), one of the U.S. metropolitan areas with the highest Chinese population [18]. It was a 2-tier process: (a) development of an KFCKD self-help booklet and (b) evaluation of patient acceptance of the booklet using content analysis of interviews. It was granted approval from the organization's Institute Review Board.

### 2.1. Booklet Development

**Figure 1:**
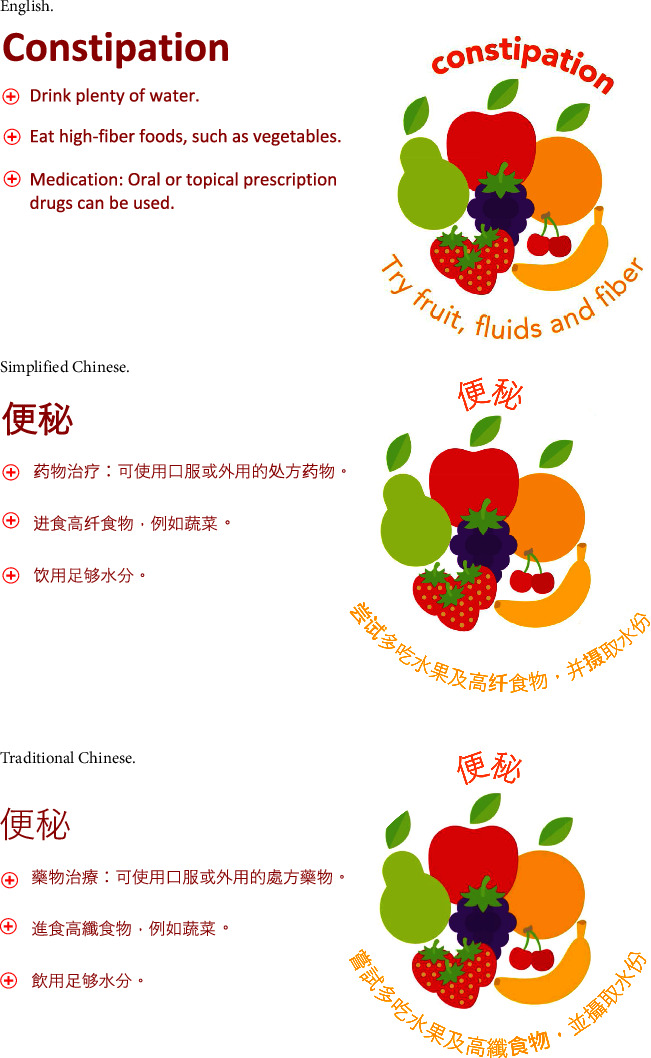
Example of strategies presented in each version of the booklet: English, simplified Chinese, and traditional Chinese (pdfs of the booklets are available for free by contacting the authors).

The booklet built on a previous study that validated the effectiveness of the strategies identified by patients to alleviate commonly occurring bothersome symptoms as described above [17] and the examples of figures from the booklet are included as Figure 1 in this publication (the complete booklets are available by contacting the author).

After confirming the symptoms and strategies to be covered in the booklet, an adapted Suitability Assessment of Materials (SAM) checklist that captures domains of message content, text appearance/typography, visuals/graphics, and layout/design [19] was used to guide the booklet content presentation. The English version of the booklet was developed first and then translated into traditional Chinese and simplified Chinese by a Chinese-English translator. All three booklet versions, as well as this study, were approved by the Institutional Review Board for our study use.

The resultant booklet was an 11-page, 4-color, letter-sized, printed booklet entitled “Self-management Strategies For Older Chinese-American Patients with Chronic Kidney Disease (CKD)”. The booklet was compiled to cover 33 strategies to ameliorate 12 symptoms including physical fatigue, trouble sleeping, pain, weak or painful arms or shoulders, itching, dizziness, trouble breathing, poor appetite, nausea and vomiting, constipation, dry eyes, and blurred vision. For each symptom, there was at least one graphic or picture illustrating one of its corresponding strategies. The recommended strategies, suggested by participants and validated by an expert panel (described above), encompassed culturally accepted practices like Tai Chi, Qi Gong, acupuncture, and Western medical remedies.

### 2.2. Evaluation of Patient Acceptance

#### 2.2.1. Patient Recruitment

We recruited patients from the pool of those who had participated in the development of the booklet and agreed to be contacted again for evaluation of the resultant booklet. The original participants were patients from a collaborative visiting nurse service provider in New York who were recruited using purposive sampling. The inclusion criteria included (a) Chinese or Chinese American living in New York City; (b) Mandarin Chinese, Cantonese Chinese, or English speaking; (c) diagnosed with KFCKD and other chronic conditions; (d) noninstitutional; (e) negative cognitive screening [20]. The patient recruitment and eligibility screening were done via telephone by a Chinese (Cantonese/Mandarin)-English investigator, a nurse practitioner with practice caring for patients in Chinese-speaking countries, as well as in the U.S. both in clinic and via in-home care, who was fluent in English, Mandarin, and Cantonese. To minimize sampling bias and to test the readability of our booklet more thoroughly, we did not exclude those who reported that they were unable to read words but allowed them to read the booklet with a caregiver. Those with hearing or visual problems were excluded.

Participants were mailed our booklet and research information sheet written in the language preference they indicated. All participant interviews were scheduled about 2 weeks after the mailing to allow adequate reading time.

#### 2.2.2. Data Collection

Data collection was done by the same investigator. In compliance with COVID-19 pandemic control measures, all data were collected over the phone. To encourage personal opinion and avoid any potential group thinking, one-to-one instead of focus group interviews were adopted. Each participant's informed verbal consent was obtained right before each interview. Also, the interviewer proceeded to interview only when the participants reported having completed reading the materials. The interviews were semistructured with a set of 4 open-ended questions including “What do you think about this booklet', “Describe how you might improve the booklet', and “How would you use this booklet' by the nurse practitioner mentioned above.

#### 2.2.3. Data Analysis

The interviews were transcribed and translated from Chinese into English by the interviewer. Then, the interviewer and two other researchers analyzed each transcript independently by the inductive thematic method [21]. Any inconsistencies in the analytic findings were discussed among the research team. All the resultant codes and themes were agreed upon by all team members.

## 3. Results

### 3.1. Participant Characteristics

Seven patients, ranging in age from 64 to 85 years old, participated in the interviews. Four participants were female and three were male. English was not their preferred language. Four participants chose the simplified Chinese booklet and the three chose the traditional Chinese version. Five interviews were conducted in Cantonese and the 2 others were in Mandarin.

### 3.2. Data Analysis Findings

Five themes about participants' acceptance of our booklet were identified: content, graphics and design, reading experience, suggestions on improvement, and health information source.

#### 3.2.1. Content

*(1) Readability*. The findings regarding booklet readability were mixed. Half of the participants found the content clear, and the provided booklet was written in their native reading language:

“I can read Chinese. I mean it's easy to understand.” (Participant E, aged 82, Simplified Chinese)

Even though they were provided with a booklet in their preferred language, some participants still found it difficult to understand the content because of factors such as age-related cognitive decline, low education level, inadequate reading comprehension skills, and presence of unfamiliar words in the text:

“It isn't that she can't read the text. She can read it but, because of her old age, she forgets what she's just read easily. You need to keep reminding her of things. For example, you need to ask her to walk and exercise a bit more, or get adequate rest as suggested here. That's all because of her old age. She can't remember things that you told her a long time ago… She's in her 80s already and there is no way she can understand all the content.” (Participant C's daughter, participant aged 80, Simplified Chinese)

Participants seemed to comprehend the content better when they were guided to read it aloud:

“It's (the booklet) about symptoms of kidney disease…It tells what is good to do and what isn't.” (Participant A, aged 81, Traditional Chinese)

#### 3.2.2. Length and Coverage

There was no information reported to be missing in the booklet. Participants who had no problems in understanding the content were generally satisfied with the booklet coverage and the length of time they needed to complete reading the booklet. However, those with difficulty in understanding the content thought the booklet provided too much information for them to process. One participant specified his desired length of booklet:

*Participant*: “There's a little bit too many (words) but it's still acceptable.” *Interviewer*: “How many pages do you think would make it easier for you?” *Participant*: “5 pages would be fine.” (Participant G, aged 73, Simplified Chinese)

*(1) Legibility*. The font size of the text was appropriate to most participants, and they were able to read the text clearly:

“I can read (the text) clearly…I even don't need wearing eyeglasses (to read).” (Participant B, aged 68, Simplified Chinese)

#### 3.2.3. Acceptance

A number of participants agreed that the content of the booklet was highly relevant to them.

“It's about my own problems!… Everything written here is about my illness.” (Participant F, aged 85, Traditional Chinese)

“I've got similar symptoms!” (Participant B, aged 68, Simplified Chinese)

High perceived relevance could help predict higher acceptability and practicability of the health advice given in the booklet:

Participant: “Yes! Yes! It's (the content) useful.” *Interviewer*: “Is there any new information about self-management skills that you feel interested in but don't know how to implement?” *Participant*: “Basically none.” (Participant B, aged 68, Simplified Chinese)

However, the extent to which the advice was accepted also varied by factors such as participants' personal habits, physical condition, self-perceived competence, and perceived usefulness of the advice. Some of the participants reported the booklet helped certain symptoms, while others found the advice superficial:

*Participant*: “I think it (the content) may be more able to address (the problems of) ordinary people. It's not quite applicable to kidney transplant patients like me… I mean it works for those usual people but not quite for those who have undergone renal dialysis and have upper and lower limb disability. For example, I have problem with my legs, while other people can walk unaided. I need to use my walking stick, you know? You haven't seen my condition:”

Interviewer: “That means you find the advice on exercise not really relevant to you?”

*Participant*: “Yes! Yes! …The arm for dialysis couldn't be touched or moved. Half of the (hand) function has been lost already…It's (the advice on exercise) not suitable to me. The only thing I can do is to take great care of myself and be careful in everything, that's it!” (Participant D, aged 64, Traditional Chinese)

“I can't say the things (symptom self-management recommendation) are practically too simple…I have kidney problem and I should avoid night food. However, I just can't do that. I'm used to have frequent, small meals even in nighttime. What suggested here is just different from my daily habits.” (Participant B, aged 68, Simplified Chinese)

*(1) Graphics and Design*. Overall, participants though it was a good idea to add pictures in the booklet (examples in Figure 1).

While there were exceptions, most of the pictures could be understood by the participants and help text comprehension:

“(It can help me to) understand (the content).” (Participant E, aged 82, Simplified Chinese) 5-3

“What's the picture of eyes about? I don't understand.” (Participant G, aged 73, Simplified Chinese)

Similarly, the color use was well accepted by the participants except a participant's family who held a different view on it:

“That might be better if it's black and white printing… She'll feel tired easily if it's too colorful.” (Participant C's daughter, participant aged 80, Simplified Chinese)

*(2) Reading Experience*. The extent of reading motivation varied among participants. Overall, most participants tended to reuse the booklet and perceived it as a good tool for reminding them of the key symptom management strategies. Also, the reading itself did not cause more physical discomforts to them:

“I'd read it again if there's something unclear. My memory isn't good… I'm not that old as the other people but my memory is worse than them, just like having dementia! I do think so!… With this booklet, I can read the information anytime and thus understand it better. You know, my memory isn't very good.” (Participant D, aged 64, Traditional Chinese)

“The more times I read, the more I can remember.” (Participant F, aged 85, Traditional Chinese)

On the other hand, some participants seemed to consider reading the booklet as a task only and did not show interest. For them, reading was a burden, resulting in their relatively low reading motivation:

“(I feel) quite tired (after reading the booklet).” (Participant G, aged 73, Simplified Chinese)

“It's only practical if I read the booklet to her. Otherwise, she'll feel it's very difficult.” (Participant C's daughter, participant aged 80, Simplified Chinese)

*(3) Suggestions for Improvement*. When being invited to provide suggestions for improving the booklet, most participants gave no specific comment or failed to articulate their views on it, except for a participant who expressed a desire for more tailor-made contents:

*Interviewer*: “In general, what areas in the booklet do you think are needed to improve?” P: “No, there's none!” (Participant B, aged 68, Simplified Chinese)

*Interviewer*: “You mean it'd be better if the booklet can give more feasible exercise advice to people with physical conditions like yours, right?”

*Participant*: “Yes! Yes! That's it!” (Participant D, aged 64, Traditional Chinese)

*(4) Health Information Source Preference*. In general, participants did not have comments on using the booklet as their source of health information. When it came to other information formats, their responses were similar:

“(I have) no comments (on using the booklet to get information about symptom management skills).” (Participant A, aged 81, Traditional Chinese)

However, some participants reported that they got the health information they needed by other means:

“The (dialysis) clinic does let us review our reports and know the foods to avoid. I just simply follow their advice.” (Participant D, aged 64, Traditional Chinese)

## 4. Discussion

This study validated the booklet as a useful source of health information in the older Chinese American KFCKD patient population. Researchers initially decided to host the one-on-one interviews using an online video conference platform, but not all the participants had the basic knowledge needed to join virtual meetings. Therefore, the traditional telephone interview method was adopted instead. From this, we deduce that currently, for our target population, the use of printed health education materials is appropriate for health information transmission in today's digital era. Because of aging- and disease-associated cognitive decline, older people, especially those with relatively low education levels, may struggle with technology application. Such technology illiteracy hinders them from accessing web-based information effectively [22]. Additionally, older people can refer to health information more easily if they have a printed booklet in hand. Even with increasing digital education formats among seniors, cultural and literacy barriers may make it so that health education booklets are likely to remain acceptable among the older in the near future [22]. Given the recommendations regarding the booklet's length and caregiver feedback on participants' memory retention, providers should emphasize to patients that the booklets are designed as reference materials. They are not meant to be read in one sitting and can be consulted as needed.

Overall, the booklet was deemed readable and relevant by most of the participants, and few suggestions were given for improvement. This is not surprising since the contents were developed in conjunction with the participants. Nevertheless, they appreciated the booklet and reported using it to refresh their symptom self-management skills. Although the target patients of the current study were all residing in New York City where access to healthcare facilities with Chinese-speaking providers was relatively high compared to other states in the U.S., they still may not always have effective physician-patient communication. Chinese American patients are inclined not to share decision-making with their healthcare providers even if given the opportunity because of their culturally based belief to not be burdensome to others, especially amongst authority figures such as physicians and nurses [12]. Additionally, older patients' decreased attention span and memory decline further impose challenges to physician-patient interaction when the medical consultation time is limited. Therefore, we suggest making our booklet available to this patient group via nephrology healthcare providers who may have additional time with patients (such as during dialysis treatments), to empower patients with better self-management skills. By providing a booklet, patients can repeatedly read the content and adjust their reading pace to their preference. Also, healthcare providers may incorporate this booklet into patient encounters, prompting patient's interest in reading the booklet, which in turn promotes more effective physician-patient interaction within the limited time of a clinical visit. This could improve the shared decision-making process and encourage the use of conservative management for KFCKD.

Even though the usefulness of our booklet was perceived as minimal by some participants, the value of this booklet in promoting the well-being of patients, even those who have trouble reading because of their physical limitations, should not be underestimated. While the current study aimed at exploring our target population's acceptance of the booklet, the role of patients' family members and caregivers in the booklet use should also be considered. Some participants needed their family members or caregivers to assist them in reading the booklet for the purposes of the interview. To a certain degree, this reflects how the booklet will be used by the target population in real situations. Readers of the booklet are not only the patients but also their family members and caregivers. While a great number of the target population are first-generation immigrants, their family members may be native-born and native in English rather than Chinese. To promote fuller use of the booklet, the language preference of the family members and caregivers should be considered during booklet distribution. An English version booklet has been developed to serve this purpose.

The credibility of health education materials is important. According to a study by Lu, Liu, and Yuan [23], Chinese people highly regard interpersonal sources such as doctors and family and friends as their health information source. This emphasizes the importance of having the health care practitioner distribute these types of materials to this population. Although our booklet was based on collective data from our pilot patient interviews, expert opinions, and current literature, some participants questioned its usefulness. To improve readers' perceived usefulness of the booklet, future iterations could include case studies or the experiences of other patients with similar symptoms.

Notably, some of the self-help strategies that were reported effective by some participants in the first phase of booklet development were rejected for the final version because they conflicted with current evidence, or their safety was unconfirmed. For example, some traditional Chinese medicines and over-the-counter Chinese herbal products, such as “Po Sum On” for dyspnea, that participants recommended did not have a sufficient evidence base, so they were not included. The deep-rooted cultural health belief of limiting moderate physical exercise in old age [24, 25] was also recommended by several participants in early phases of the study; this belief was excluded because it is counter to current evidence-based practice [26, 27]. This exclusion may have made the booklet seem not culturally relevant from the reader's perspective. Explanations of harmful or non-evidence-based treatments were excluded to maintain the booklet's clarity and brevity.

### 4.1. Limitations

Sampling bias limits the generalizability of our findings to all patients in the target population. Patients who had difficulty joining phone interviews because of hearing deficits were not included in our study. These patients generally experience even more health inequity than others in our target population because their hearing problems may hinder them from communicating effectively with their healthcare providers and caregivers. This communication barrier may also limit their sources of health information because information in audio formats. In such cases, printed materials such as booklet may be the only feasible source for obtaining information about self-symptom management skills. As hearing was the second most reported disability in older patients with chronic kidney disease stages 3 and 4 [28], their perception of this booklet should not be disregarded. In-person patient interviews may help in gathering additional opinions of the booklet.

The use of phone interviews in data collection was another limitation of our study. Originally, face-to-face interviews were planned, but because of the COVID-19 pandemic, the study was confined to telephone interviews. Face-to-face interviews would have been preferable to allow the researcher the opportunity to interpret participants' facial expressions and nonverbal cues during data collection. Face-to-face interviews also allow more efficient researcher-participant interaction.

Currently, the different language versions are separate. Future iterations could include a version that combines all three texts (English, simplified Chinese, and traditional Chinese). Furthermore, it was outside the scope of this study to evaluate the caregiver response to the booklet, but this could be considered in future studies.

Finally, investigating the degree to which a patient accepts the booklet in a research setting was itself a limitation. Participants might feel embarrassed about critiquing the booklet during formal interviews. Such feelings of embarrassment might be amplified in the Chinese population where many people are accustomed to accepting without questioning. This phenomenon is particularly true in the physician-patient relationship: Chinese immigrants tend not to be involved in their health decision-making [12], thus giving up their right to express their opinion even when given opportunities.

### 4.2. Implications for Nursing and Health Policy

Improving cross-cultural interactions should be a goal for nurses and nurse managers as patient diversity continues to increase. Previous work has demonstrated the effectiveness of equity-based healthcare in improving patient outcomes for marginalized groups. In a longitudinal study, Ford-Gilboe et al. [29] found that patients who received such care reported feeling more confident in their care and ability to manage symptoms. By providing them with this booklet and adapting to patients' preferences in their preferred language, providers may have improved concordance with patients and older Chinese American KFCKD patients may feel more confident in their ability to manage common symptoms at home.

As previously discussed, the cultural tendency of these patients to avoid being burdensome makes it especially important to provide them with the tools to self-manage their symptoms. Because we created this symptom management booklet as a general guideline for all patients, presenting the booklet without further instruction would be insufficient in a clinical setting. For example, while the booklet says, “increase fluid intake,” the provider should give instructions on the daily goal for fluid intake in accordance with the patient's current health diagnoses and symptoms. Clinicians using this booklet should be certain to personalize the booklet and provide specific details in reference to the patient's situation. Policies regarding patient education should incorporate culturally sensitive materials in formats acceptable to those with limited English-language or technological literacy.

## 5. Conclusions

This study demonstrated the general acceptance of the printed booklet by older Chinese American KFCKD patients. We suggest using the booklet as supplementary health education material in clinical settings, including use among patients' family members and caregivers to maximize its effectiveness in helping the target population handle troublesome symptoms in the home setting. To improve the cultural sensitivity of the booklet, further studies of patient health behavior in this population are needed to incorporate cultural practices into evidence-based materials.

## Data Availability

Data from the original surveys were uploaded and may be accessed through the cdRNS at https://cdrns.nih.gov. Due to the personalized nature of the interviews that would make them impossible to deidentify, interview data are not available. The completed booklets can be obtained by emailing the corresponding author.
